# Complete mitochondrial genome of *Stictochironomus akizukii* (Tokunaga) (*Chironomidae, Diptera*) assembled from next-generation sequencing data

**DOI:** 10.1080/23802359.2020.1750320

**Published:** 2020-06-05

**Authors:** Kiyun Park, Hyunbin Jo, Bohyung Choi, Ihn-Sil Kwak

**Affiliations:** a Fisheries Science Institute, Chonnam National University, Yeosu, Republic of Korea; b Faculty of Marine Technology, Chonnam National University, Yeosu, Republic of Korea

**Keywords:** Mitochondrial genome; Chironomidae

## Abstract

The complete mitochondrial genome of *Stictochironomus akizukii* (Tokunaga) was sequenced. The circular mitochondrial genome is 15,052 bp and consists of 13 protein-coding, 3 ribosomal RNAs, and 22 transfer RNA genes (GenBank accession no. MT185679). Results of maximum likelihood analysis showed that this species clustered with other species of the family Chironomidae. This study will contribute to the phylogenetics of genus *Stictochironomus* and the other genera of Chironomidae.

*Stictochironomus* is a genus of non-biting midges with strongly marked wings and legs in the subfamily of the bloodworm family Chironomidae. *Stictochironomus akizukii* (Tokunaga) live in sand and other sediments in a variety of unpolluted fresh water habitats (Kiyashko et al. 2004). *S. akizukii* (Tokunaga) could be a good species for monitoring freshwater quality. In the Chironomidae, a molecular barcoding approach of mitochondrial region has been reported as the most partial cytochrome c oxidase subunit I (COI) gene genome to discriminate among Chironomidae species (Kim et al. 2016; Montagna et al. 2016). Furthermore, complete sequences of mitochondrial genomes (mitochondrial DNA [mtDNA]) of *S. akizukii* (Tokunaga) were not determined. In this study, we described a first complete mtDNA of *S. akizukii* (Tokunaga), assembled using next-generation sequencing. Specimens of *S. akizukii* (Tokunaga) were collected from the Seomjin River, South Korea (34°59′11″N, 127°46′38″E) on April 2019. Genomic DNA was extracted using a DNeasy tissue kit (Qiagen, Valencia, CA). It was stored at Specimen Museum of Fisheries Science Institute, Chonnam National University (accession number CNUISI-010905201). The library preparation and DNA sequencing (100 bp mate pairs with different insert sizes, Illumina HiSeq4000) were performed at Macrogen Inc., (Seoul, Korea). The genome was assembled using the MEGA-X software (Kumar et al. 2018). The annotated mitochondrial genome sequence of *S. akizukii* (Tokunaga) is available at the National Center for Biotechnology Information (NCBI) database (GenBank accession number MT185679).

The complete sequence of the mtDNA of *S. akizukii* (Tokunaga) is 15,052 bp and was comprised of 13 protein-coding genes, 3 rRNAs, and 22 tRNAs. Nucleotide distribution is as the following: A – 38.7%, C – 13.2%, G – 11.9%, and T – 36.2%, and the GC content is relatively low 25.1%. Phylogenetic analysis was performed to examine the relationships of *S. akizukii* (Tokunaga) and other ten closely related dipterans based on the mitochondrial genomes by MEGA-X (Figure 1). The resulting phylogenetic tree showed that *S. akizukii* (Tokunaga) forms a clade with *Chironomus tepperi* and *Polypedilum vanderplanki* in Chironomidae.

**Figure 1.**
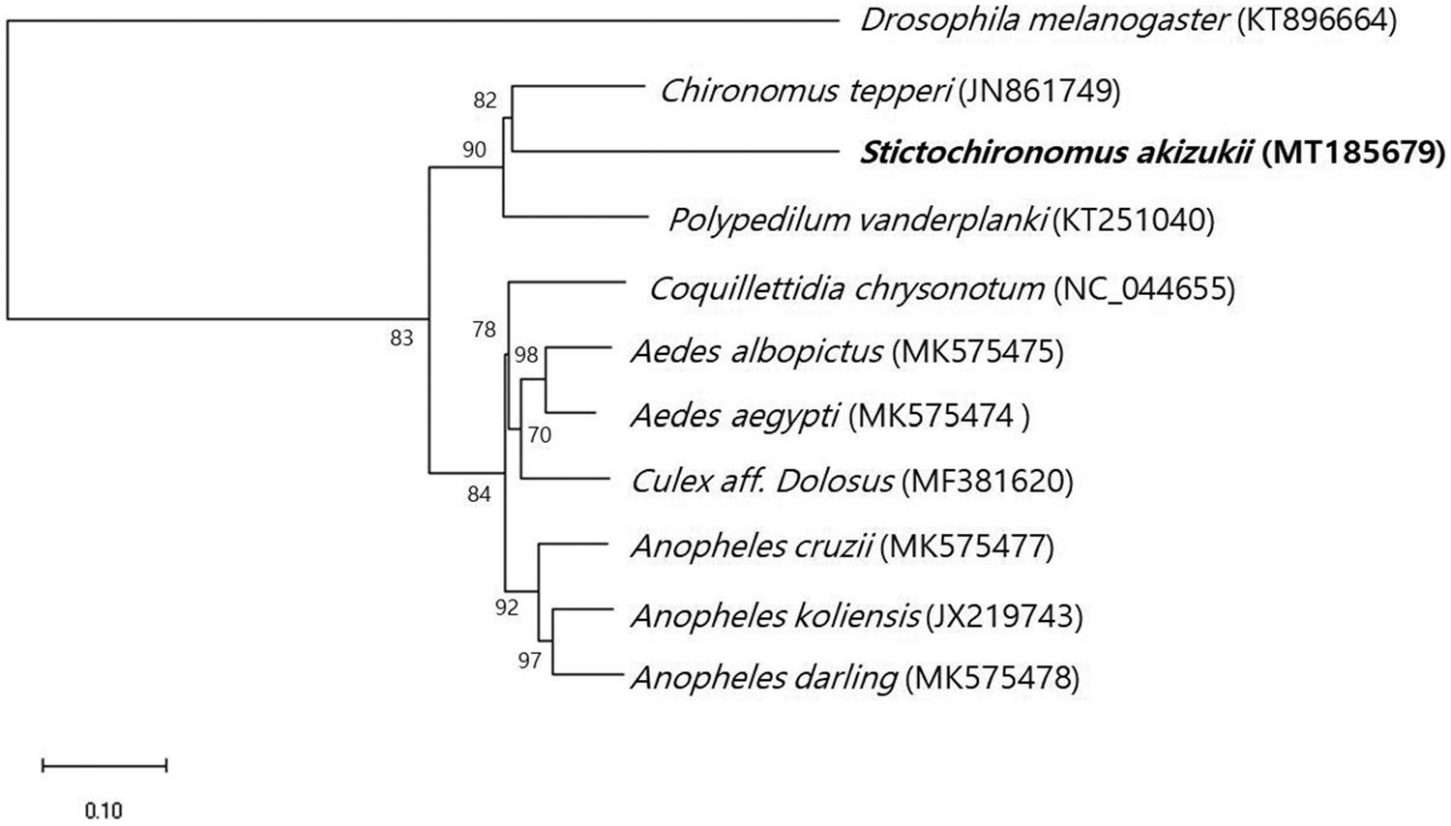
Maximum-likelihood tree is based on 11 Dipteran mitochondrial genomes. All the bootstrap values after 1000 iteration are indicated at the nodes.
